# Health‐related stigma of noncommunicable neurological disease in rural adult populations: A scoping review

**DOI:** 10.1111/hsc.12694

**Published:** 2018-12-12

**Authors:** Valerie L. Elliot, Debra Morgan, Julie Kosteniuk, Amanda Froehlich Chow, Melanie Bayly

**Affiliations:** ^1^ Canadian Centre for Health and Safety in Agriculture University of Saskatchewan Saskatoon Saskatchewan Canada

**Keywords:** adult, neurological disease, rural, stigma

## Abstract

Stigma is a widely recognised public health issue. Many people with neurological disease and their families experience stigmatisation, adding to their burden of illness. Rural populations are typically small, lack anonymity, and often have a higher proportion of older adults with inadequate access to specialised services and resources. Although generally isolated, rural areas can offer benefits such as a sense of familiarity and interconnectedness. The purpose of this scoping review was to map the existing evidence on stigma associated with non‐communicable neurological disease in rural adult populations and identify key findings and gaps in the literature. Our literature search of peer‐reviewed English language articles published from 1 January 1992 to 22 June 2017 was conducted across five databases yielding 8,209 results. After duplicate removal, pairs of reviewers independently screened 6,436 studies according to inclusion criteria developed a priori; 36 articles were identified for inclusion in this review. Study characteristics were described and illustrated by frequency distribution, findings were grouped thematically, and each of the five types of stigma were identified (social, self, health professional, associative, structural). Four factors influencing stigma (knowledge, familiarity, beliefs, and rurality) and four overarching stigma‐related themes (concealment; exclusion; disempowerment, discrimination, and unequal opportunities; and issues related to healthcare systems and providers) emerged. In urban‐rural comparison studies, rural residents were generally less knowledgeable about the neurological disease and more stigmatised. The impact of other factors (i.e., gender, age, and education) on stigma varied and are stated where associations were reported. Three main gaps were identified including: low attention to stigma related to neurological diseases other than epilepsy, limited cross‐cultural comparisons of stigma related to neurological disease, and inclusion of gender as a variable in the analysis of stigma‐related outcomes in only half of the reviewed studies. Further research is recommended.

**What is known about this topic**

- Health‐related stigma increases the overall burden of illness.
- The stigma of neurological disease is a widely recognised global health issue.
- People living in rural areas experience unique circumstances that warrant particular consideration.

**What this paper adds**

- Although rural areas have characteristics that can both reduce and increase health‐related stigma, in general our findings indicated poorer stigma‐related outcomes among those living rurally compared to urban.
- Key gaps in the literature, factors that influence stigma, and overarching stigma‐related themes were identified.
- These findings can be used to inform future research, policy, and intervention strategies designed specifically for rural areas aimed at reducing the stigma of neurological disease and the negative consequences, thereby improving the lives of this population and their families.

## 1 INTRODUCTION

The negative physical and psychological effects of health‐related stigma that contribute to the burden of illness are well known (Hatzenbuehler, Phelan, & Link, 2013; Scambler, 2009; Weiss, 2006; World Health Organization, 2006). To address existing vague and individualistic definitions, Link and Phelan (2001) developed a useful framework to portray the complexities of stigma that occur as an interplay of “labelling, stereotyping, separating, status loss and discrimination” (p. 377) within a situation of power, that permits development of these actions to take place. Following the publication of Erving Goffman's seminal book on stigma in 1963, the topic has been a growing concern across multiple disciplines (Goffman, 1963). There has been increasing recognition of the role stigma plays in relation to specific diseases and the adverse social, economic, and health effects that have rendered it a public health issue (Hatzenbuehler et al., 2013; Scambler, 2009; Weiss, 2006; WHO, 2006). Current variations in conceptualisations, definitions, types, and degrees of stigma exist and appear to stem from both the situational and multidisciplinary application of the term, in addition to its being comprised of multiple interrelated components (Link & Phelan, 2001).

The association between stigma and neurological disease is widely recognised as a global health issue for many people with neurological diseases and their families (WHO, 2006). In 2015, neurological diseases were identified as the main cause of disability‐adjusted life years and the second main cause of death globally (GBD, 2017). Neurological disease can be classified as either communicable (infectious) or noncommunicable (chronic). From 1990 to 2015, the global burden (premature mortality and morbidity) of communicable neurological disease has decreased, whereas the global burden of noncommunicable neurological disease has continued to rise (GBD, 2017). This is in line with other global communicable ‐ noncommunicable disease trends in general (GBD, 2016) and has been largely attributed to an ageing population and population growth in general (GBD, 2016).

Although the global population, including the older population, has been rising faster in urban areas relative to rural settings, almost half (46%) of the world's population continues to live in rural locations (UN, 2014). People living in rural areas experience unique circumstances that can present both beneficial and challenging influences on health, including health‐related stigma (Gessert et al., 2015). Thus, gaining a better understanding of stigma associated with neurological disease in rural adult populations is warranted.

For this review, five types of stigma are relevant: (a) social stigma, (b) self‐stigma, (c) stigma by association, (d) structural stigma, and (e) health professional stigma. According to Bos, Pryor, Reeder, and Stutterheim (2013) stigma can be viewed as taking place on three levels: societal, interpersonal, and individual. Bos et al. (2013) also offer an enhanced description of four interconnected types of stigma identified in the conceptual model of Pryor and Reeder (2011) as public stigma, self‐stigma, structural stigma, and stigma by association. Social or public stigma represents the central stigma type and refers to the stigmatising responses of others towards the person who has a stigmatised condition (Bos et al., 2013). Self‐stigma refers to the perceptions and feelings of stigma that are internalised by the person with a stigmatised condition, due to their awareness and experience of social stigma towards them (Corrigan, 2004; Corrigan, Larson, & Rüsch, 2009; Crocker, 1999). Stigma by association refers to the social stigmatisation of individuals simply by virtue of being associated with a stigmatised person (Mehta & Farina, 1988; Neuberg, Smith, Hoffman, & Russell, 1994; Pryor & Reeder, 2012; Werner & Heinik, 2008). Structural stigma refers to the systemic, institutional, policy, and societal structures that fundamentally and disproportionately limit the rights and freedoms of certain groups of people, such as people who have a stigmatised condition (Corrigan, Markowitz, & Watson, 2004; Hatzenbuehler, 2016; Link & Phelan, 2001). Lastly, although the phenomenon of health professional stigma is an understudied type of stigma that could be considered part of the social stigma realm, their position and duty of care places them in a unique situation of importance (Ahmedani, 2011). The attitudes and beliefs of healthcare professionals about certain diseases and toward their patients inarguably play an important role in health‐related stigma.

The study objectives were to: map the existing evidence of stigma associated with noncommunicable neurological disease in rural adult populations; identify key findings and gaps in the literature; and make recommendations for future research. The findings from this review contribute to the existing knowledge of stigma related to neurological disease from a rural‐centred perspective.

## 2 METHODS

The protocol used to conduct this scoping review is based on the Arksey and O'Malley (2005) five‐step framework which included (a) identifying the research questions, (b) identifying the relevant studies, (c) study selection, (d) data charting, and (e) collating, summarising, and reporting the results. A collaborative research team approach was used, consisting of all authors (VE, JK, AFC, MB, DM), in the iterative process of developing the research questions and search strategies, and creating the data extraction form.

### 2.1 Stage 1: Identifying the research questions

The purpose of this review was to gain a better understanding of stigma related to noncommunicable neurological disease in rural adult populations and the consequences of this stigmatisation, to potentially inform and assist with future stigma reduction efforts. We focused on three main questions.

1. What are the main aspects/issues of stigma associated with noncommunicable neurological disease in rural adult populations, specifically:
  - What types of stigma exist?
  - What are the main factors associated with stigma and what is their effect on stigma?
  - What are the main themes related to stigma?

### 2.2 Stage 2: Identifying the relevant studies

Search strategies were developed with the guidance of a university health sciences librarian. A broad search strategy was designed to capture all literature within each of the five databases searched (MEDLINE, PsycINFO, EMBASE, CINAHL, and SocINDEX) that pertained to both “stigma” and “rural”. The intent of this comprehensive approach was to avoid placing narrower restrictions on our search, thereby prematurely limiting our pool of evidence from which to screen. Thus, a keyword search using the multipurpose (.mp) combined set of fields (title, abstract, subject or MESH headings) for all databases included the following search terms: (rural*) AND (stigma* OR attitude OR prejudice* OR stereotyp* OR discrimination or perception*). Search strategies were customised to the specific requirements of each database. The search was limited to English language studies published over a 25‐year period, from 1 January 1 1992 to 22 June 2017.

### 2.3 Stage 3: Study selection

**Figure 1:**
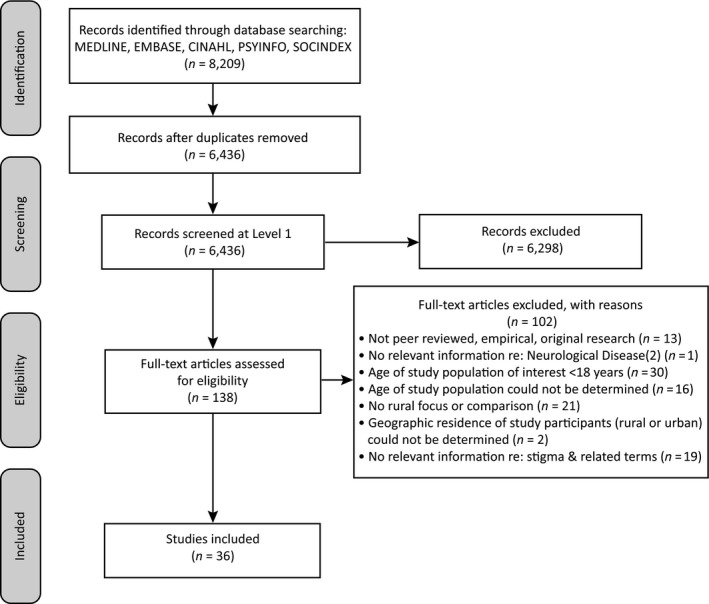
PRISMA flow diagram of the study selection process (Moher, Liberati, Tetzlaff, & Altman, 2009)

The search terms yielded 8,209 results across five databases (Figure 1). The studies were exported from the databases into EndNote (Clarivate Analytics, Philadelphia, United States) and duplicate publications removed. Studies were then exported to DistillerSR (Evidence Partners, Ottawa, Canada). After a second deduplication process, 6,436 studies remained to be assessed for inclusion regarding relevance to the research questions.

All types of research designs were considered for inclusion. Additionally, the inclusion criteria (Table 1) required that the studies address the following: (a) the neurological disease was noncommunicable and was listed in the National Institute of Neurological Diseases and Stroke (NINDS) (Disorders, n.d.), (b) the study focus was on adults aged 18 years or over with a neurological disease, and (c) there was sufficient relevant information regarding stigma (or associated terms) related to neurological disease. Studies that did not focus on or make a comparison to rural participants were excluded. Only published, original, peer‐reviewed research articles were considered for inclusion.

**Table 1:** Inclusion and exclusion criteria

Inclusion criteria	Exclusion criteria
Peer‐reviewed, original research only	Letters to the editor, opinion letters, commentaries, dissertations, study protocols reviews, policy papers, reports, book chapters, and all other nonpeer‐reviewed documents
Publications in English language only	Publications written in a language other than English
Published from 1992 to 22 June 2017	Published outside of 1992 to 22 June 2017
Addresses main topic (stigma [or related terms] re: noncommunicable neurological diseases in rural adult populations)^a^	Main topic not addressed (no relevance to stigma [or related concepts] re: neurological diseases in rural adult populations^a^
Study population aged ≥18 (study focus on adults with neurological disease)	Study population <18 (study focus on children with neurological disease)
Study participants may include patients (self), informal caregivers/family, health professionals, community members	Studies that do not focus on or make a comparison to rural population

a Neurological Diseases identified as per those recognized by the National Institute of Neurological Diseases and Stroke (NINDS).

Screening forms were created and piloted in DistillerSR to perform the screening processes at two levels. At the title and abstract level, two authors independently screened studies for inclusion. The first author screened all studies and three coauthors each screened one‐third of all studies. Unresolved conflicts at this level were included for screening at the full‐text level. Two reviewers independently screened studies for inclusion at the full‐text level (*n* = 138) and unresolved conflicts were resolved by the second author. Thirty‐six studies were included for synthesis.

### 2.4 Stage 4: Data charting

A data chart was piloted by three reviewers to extract and summarise the relevant information from each study. The first author extracted study characteristics and findings from each of the 36 studies. Data extracted included: author, year, country, objective, population, and design. Additional study information and findings extracted were: neurological disease, definition of rural, definition of stigma, stigma measure, main findings related to the stigma of neurological disease, and the type of stigma identified.

### 2.5 Stage 5: Collating, summarising, and reporting the results

**Figure 2:**
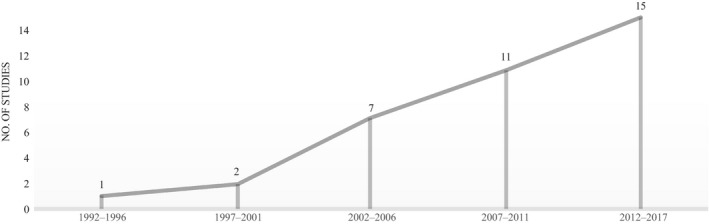
Included studies by publication year

**Figure 3:**
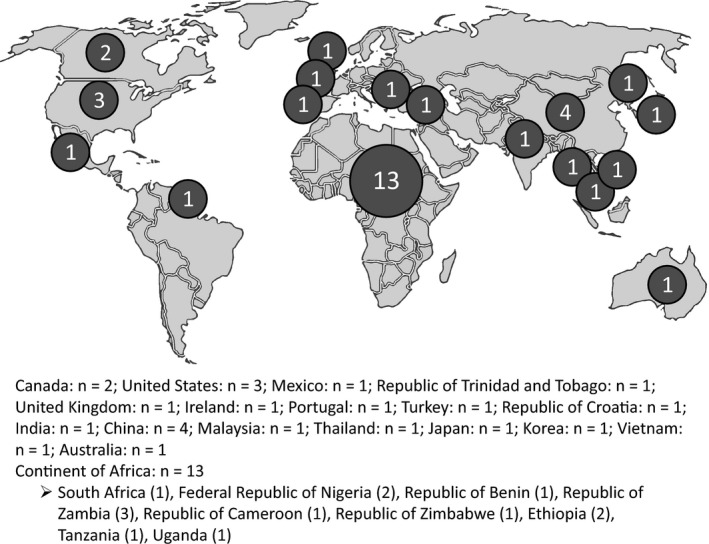
Included studies by geographic location (*n* = 36)

**Figure 4:**
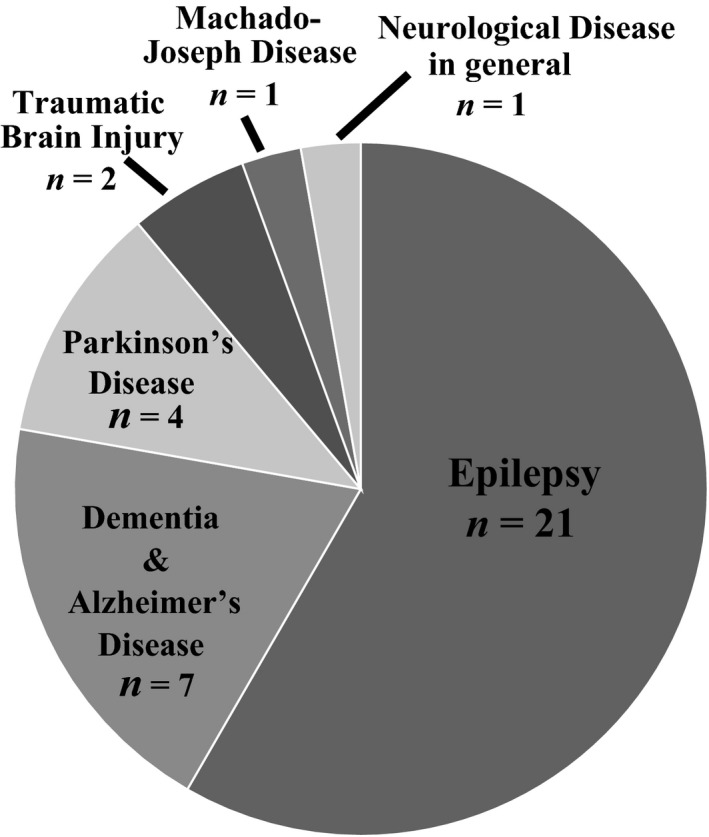
Included studies by neurological disease (*n* = 36)

The extracted data for all 36 studies were collated by year of publication, country, and neurological disease, which are illustrated by frequency distribution (Figures 2, 3, 4, respectively). Analysis of the study findings identified four major factors influencing stigma and four overarching themes. For a brief description of themes and studies itemised by theme, refer to Table 2.

**Table 2:** Stigma‐related themes and itemized studies

Main stigma‐related themes	Theme description	Theme identified in studies
1. Concealment	Attempts to hide the ND Often associated with fear of displaying physical manifestations of disease	*n* = 11 Alston, Jones, and Curtin (2012); Arai, Sugiura, Miura, Washio, and Kudo (2000); Burgener et al. (2015); Guo et al. (2012); McQueen et al. (1995); Morgan et al. (2002); Rafael et al. (2010); San‐Juan et al. (2015); Tiamkao et al. (2013); von Gaudecker et al. (2017); Yang et al. (2011)
2. Social exclusion	By self and/or by others Of self and/or of others (i.e., friends/family) Often associated with fear (e.g., of contagion or of disease manifestations)	*n* = 23 Alston et al. (2012); Bain et al. (2013); Birbeck et al. (2006); Burgener et al. (2015); Farmer et al. (2003); Forbes et al. (2011); Guo et al. (2012); Hsiao et al. (2015); Klepac et al. (2007); Kim et al. (2003); McQueen et al*.* (1995); Morgan et al. (2002); Mshana et al. (2011); Mugumbate (2013); Neni et al. (2010); Ojinnaka (2002); Osungbade et al*.* (2011); Rafael et al. (2010); Tiamkao et al. (2013); Tuan et al. (2007); von Gaudecker et al. (2017); Yang et al. (2011); Youssef et al. (2009)
3. Disempowerment, discrimination, unequal life opportunities	Loss of power, control, choices, and chances (e.g., decision‐making, finances, employment, marriage) Includes prejudice/intolerance	*n* = 21 Atadzhano et al*.* (2006); Bain et al. (2013); Birbeck et al. (2006); Deresse et al*.* (2016); Farmer et al. (2003); Forbes et al. (2011); Guo et al. (2012); Hsiao et al. (2015); Kaddumukasa et al. (2015); Kartal et al. (2016); Kim et al. (2003); McQueen et al*.* (1995); Mshana et al. (2011); Mugumbate et al*.* (2013); Neni et al. (2010); Paúl et al. (1999); Rafael et al. (2010); Tiamkao et al. (2013); Tuan et al. (2007); Yang et al. (2011); Youssef et al. (2009)
4. Healthcare systems, services, and providers Availability and accessibility Healthcare provider beliefs	Lack of available, accessible healthcare systems, services and providers and related issues (e.g., cost, travel) healthcare provider lack of time (to address complexities of certain NDs), and beliefs (e.g., relative priority)	*n* = 9 Birbeck et al*.* (2002); Cahill et al. (2008); Guo et al. (2012); Hsiao et al. (2015); McQueen et al*.* (1995); Mshana et al. (2011); Paúl et al. (1999); Stansbury et al. (2010); Yang et al. (2011)

## 3 FINDINGS

### 3.1 Study characteristics

Study characteristics including the publication author, year, and country, in addition to study methods, sample, and stigma measures are presented with the main stigma‐related findings and stigma types in Table 3. The 36 studies were published from 1995 to 2017, with an increase in the number of studies over time (Figure 2). Included studies were comprised of geographical locations from across the globe (Figure 3).

**Table 3:** Study characteristics and main stigma‐related findings

Author, Year, Country	Methods, Sample	Stigma measure(s)	Main stigma‐related findings	Type of stigma identified in findings
Epilepsy (*n* = 21)				
McQueen et al*. *(1995), South Africa	Methodology: Qualitative Semistructured interviews Sample:16 rural adults with diagnosed epilepsy Gender: 12 male, 4 female Age: 20–50 years (mean: 35 years)	No explicit measure provided.	Lack of accurate epilepsy knowledge Lack of available access to affordable healthcare and services Concealment Reduced service use Employment discrimination Dependency on others Social exclusion and self‐isolation	Social Self Associative Structural
Ojinnaka (2002), Federal Republic of Nigeria	Methodology: Quantitative Cross‐sectional survey Sample: 125 rural teachers Gender: 54 male, 71 female Age: 25+years (78% between 25 and 40 years)	No direct measure of stigma but assesses related aspects including: “Knowledge, beliefs, attitudes, and experience”	Lack of accurate epilepsy knowledge Less educated associated with increased stigma Cultural influence on inaccurate knowledge (e.g., caused by “spirits”), associated with more stigmatization Social exclusion; associated with false beliefs (e.g., “contagious”)	Social Structural
Atadzhano et al*. *(2006), Republic of Zambia	Methodology: Quantitative Cross‐sectional survey Sample: 225 clerics (73.5% rural, 26.5% urban) Gender: 77.9% male, 22.1% female Age: 18–76 years (mean 40.7 years)	No direct measure of stigma but assesses Related aspects including: "Knowledge, tolerance, and familiarity/experience”	Rural compared to urban Rural less tolerant than urban Across both urban/rural High familiarity Lack of accurate epilepsy knowledge Low tolerance, associated with being younger, less educated	Social Associative
Birbeck et al. (2006), Republic of Zambia	Methodology: Quantitative Cross‐sectional survey Sample: 159 teachers (64.2% urban, 35.8% rural) Gender: 64.2% male, 35.8% female Age: 19 to 62 years (mean 35.1 years)	No direct measure of stigma but assesses related Aspects including: “Personal familiarity/experience with epilepsy, as well as knowledge and tolerance”	Rural compared to urban Rural less familiar, less knowledgeable, and less tolerant than urban Across both Urban/Rural More accurate knowledge associated with more familiarity and more tolerance Cultural influence on inaccurate knowledge (e.g., “witchcraft”), associated with more stigmatization Social exclusion	Social Associative Structural
Rafael et al. (2010), Republic of Benin	Methodology: Mixed methods Cross‐sectional survey Semi‐structured interviews Sample: 80 rural adults with epilepsy Gender: 40 male, 40 female Age: 18–76 years (mean 35.2 years)	Two direct measures: Developed a Benin‐context version of the Explanatory Model Interview Catalogue (EMIC) Jacoby's 3‐item stigma scale Two assessments of stigma‐related aspects: Sociocultural, within the EMIC framework Psychological, depression/anxiety ‐ Goldberg Anxiety and Depression 18‐item Scale	Concealment Reduced life opportunities 68% experienced stigma Dependency on others Social exclusion Cultural influence on inaccurate knowledge (e.g., caused by “spirits”) associated with more stigmatization Positive association among anxiety and depression and stigmatization More males with epilepsy than females never married Perceptions of stigma were similar for both males and females	Social Self
Osungbade et al. (2011), Republic of Nigeria	Methodology: Quantitative Cross‐sectional survey Sample: 365 rural adults without epilepsy Gender: 235 male, 130 female Age: 18–74 years (mean age 42 years)	No direct measure of stigma but assesses related aspects including familiarity, knowledge, and beliefs	High familiarity but low accurate knowledge Cultural influence on inaccurate knowledge (e.g., “demonic possession”) associated with more stigmatization Social exclusion; associated with false beliefs (e.g., “contagious”) Infectious belief associated with increased stigma Females (especially over age 30 years) were more knowledgeable about epilepsy than men	Social Self
Bain et al. (2013), Republic of Cameroon	Methodology: Quantitative Cross‐sectional survey Sample: 505 rural community members Gender: 273 male, 232 female Age: 18 years and older	No direct measure of stigma but assesses related aspects including familiarity, knowledge, beliefs, and practices	High familiarity with epilepsy Stigmatization positively associated with being older, being female, being less educated and less knowledgeable about epilepsy Cultural influence on inaccurate knowledge (e.g., “witchcraft”) associated with more stigmatization Social exclusion; associated with false beliefs (e.g., “contagious”; “madness” Inaccurate knowledge positively associated with discrimination and reduced life opportunities	Social Associative Structural
Mugumbate et al*. *(2013), Republic of Zimbabwe	Methodology: Mixed Methods Cross‐sectional structured interview questionnaire Focus groups (2) Sample: 100 rural adults diagnosed with and receiving treatment for epilepsy (questionnaire) and 20 rural health workers (focus groups) Gender: 40 male, 60 female Age: 22–65 years (mean age 37.7 years)	No direct measure of stigma but qualitatively and quantitatively assesses related aspects including beliefs, perceptions, knowledge	High inaccurate knowledge and beliefs (e.g., “contagious”), positively associated with more stigma Cultural influence on inaccurate knowledge (e.g., “punishment for sins”) associated with more stigmatization and traditional care‐seeking Social exclusion; associated with false beliefs (e.g., “contagious”; “madness”) Reduced life opportunities	Social Self
Deresse et al. (2016), Ethiopia	Methodology: Quantitative Cross‐sectional survey Sample: 1,316 randomly selected adults (660 urban, 656 rural) Gender: 612 male (334 rural), 701 female (321 rural) Age: 18–82 years (mean age 38 years)	No direct measure of stigma but assesses related aspects including knowledge, attitudes, beliefs, and practices	Familiarity positively associated with accurate epilepsy knowledge Rural lack of knowledge positively associated with increased stigmatization Reduced life opportunities are greater for rural than urban Discrimination Cultural influence on inaccurate knowledge (e.g., (“evil spirits”) associated with more stigmatization and traditional care‐seeking	Social Associative
Tegegne et al. (2016), Ethiopia	Methodology: Quantitative Cross‐sectional survey Stigma Scale Sample: 415 adults (351 urban, 64 rural) diagnosed with and receiving treatment for epilepsy Gender: 229 male, 186 female Age: 18 years and older	Jacoby's 3‐item Stigma Scale	Note: Majority Urban (18.2% Rural) Rural almost 2x more likely to experience stigma than urban Overall (Rural/Urban) Stigmatization positively associated with being divorced/widowed, lower income, frequent seizures, epilepsy drugs side effects, and rural residence No difference in stigmatization across gender/occupation/education/religion/ethnicity	Social Self
Kim et al. (2003), Korea	Methodology: Quantitative Cross‐sectional surveys (2) 1 prior to four‐year educational campaign 1 after four‐year educational campaign Sample: Survey #1:881 rural adults Survey #2 (post‐education campaign): 715 rural adults Survey #1 & #2 (post‐education campaign): 418 Gender: 40% male, 60% female Age: Over age 19 years	No direct measure of stigma but assesses related aspects including familiarity, understanding, and attitudes	High familiarity Rural more negative attitudes towards epilepsy versus urban (associated with false belief as untreatable) Cultural influence on inaccurate knowledge (e.g., (“divine punishment”) associated with more stigmatization, traditional care‐seeking, being female, older, and less educated Prejudice/Discrimination Reduced life opportunities Social exclusion Slight significant changes after four‐year educational campaign: reduction in belief of “divine punishment” as cause and traditional medicine as better	Social Associative
Tuan et al. (2007), Vietnam	Methodology: Quantitative Cross‐sectional survey Sample: 2005 randomly selected rural adults Gender: 933 male, 1,072 female Age: 19–71 years	No direct measure of stigma but assesses related aspects including knowledge, familiarity, attitudes, and practices	Living in mountain regions (vs. plains), being single, female, and younger were all associated with being less familiar with epilepsy More familiarity with epilepsy associated with less negative attitudes and stigmatization Social exclusion, associated with inaccurate knowledge/beliefs about cause (e.g., “insanity” Reduced life opportunities	Social Self Associative Structural
Youssef et al. (2009), The Republic of Trinidad and Tobago	Methodology: Quantitative Cross‐sectional survey Stigma Scale of Epilepsy Sample: 355 full‐time university students (99 City, 179 Subrural, 77 rural) Gender: 255 male, 200 female Age: 18–50 years (mean age 21.6 years)	Standardized Questionnaire included Stigma Scale of Epilepsy Other related aspects were also assessed including knowledge, attitudes, and perceptions	Rural (and mixed ethnicity) experienced less stigmatization in general versus city or subrural Across Rural/Subrural/City Hindu perceived more stigmatization and had less positive attitudes overall than Christian or Muslim Low cultural influence on inaccurate knowledge (regarding cause, only 1% “demonic” and 5% “insanity”) High familiarity with epilepsy and most would seek medical professional care (vs. traditional) for epilepsy High familiarity positively associated with positive attitudes Discrimination, social exclusion, reduced life opportunities Being employed, more educated, and higher income, positively associated with more positive attitudes	Social
Neni et al. (2010), Malaysia	Methodology: Quantitative Cross‐sectional survey Sample: 615 rural adults Gender: 43.4% male, 56.6% female Age: 18–98 years (mean age 41.6 years)	No direct measure of stigma but assesses related aspects including awareness, knowledge, and attitudes	Most (94%) were familiar with epilepsy but over half (54%) did not know etiology Knowledge of etiology: 42% hereditary; 5% contagious (60% reported it was not evil spirits or demonic possession) 60% reported epilepsy to be fatal and 47% as curable Over half (50.9%) correctly recognized as not a mental disease More positive attitudes associated with: Higher levels of education and income, and being employed Reduced life opportunities; prejudice Social exclusion No significant difference between knowledge, attitudes, and awareness for males vs. females (overall, both scored best for attitudes and worst for awareness)	Social Self
Yang et al. (2011), China	Methodology: Qualitative Semistructured interviews Focus groups (8) Sample: 93 adults working in “public positions and the institutional sphere” (47 urban, 46 rural) Gender: 31 male (20 rural), 62 female (29 rural) Age: ≥45 (*n* = 32), 35–45 (*n* = 25), <35 (*n* = 36)	No direct measure of stigma but qualitatively assesses related aspects including knowledge, attitudes, and beliefs	Rural/Urban differences Rural had more negative attitudes than urban and were more likely than urban to perceive epilepsy as “frightening” Rural (and men) more accepting of disease concealment vs. urban Rural were more concerned than urban about marriage prospects (especially for younger people with epilepsy) Rural respondents were more concerned with feasibility of treatment (costs for epilepsy treatment vs. that of other serious diseases) Rural respondents were more concerned with psychosocial issues for people with epilepsy versus other serious diseases Across both rural and urban: Social exclusion Concealment Reduced life opportunities Overall, more positive attitudes by those with medical background Many health professionals reported that people with epilepsy have an “epileptic character” with negative changes in personality and behaviour and reported the disease as “worse than other serious diseases”; concern with stigmatization Fewer men than women reported belief of epilepsy as worse relative to other diseases and focused more on physical health issues; women were more focused on the uncontrollability, unpredictability, stigma, and burden of care on others Fewer women than men reported belief of epilepsy as better than other diseases Younger people more concerned with physical issues; older people more concerned with social factors and stigma	Social Self Health professional
Guo et al. (2012), China	Methodology: Qualitative Individual in‐depth interviews (people with epilepsy) (*n* = 70) Focus Groups (others) (*n* = 36) Sample: 106 rural people (24 with epilepsy, 24 family members, 18 doctors, 4 employers, 6 community leaders, 12 teachers, 12 neighbours) Gender: People with epilepsy: 13 male, 11 female Doctors: (8 male, 16 female) Nurses: 0 male, 6 female All other groups, gender not specified Age: People with epilepsy: aged 14–19 years (*n* = 5), aged 20–35 years (*n* = 7), aged 36–59 years (*n* = 10), ≥60 years (*n* = 2) Doctors: aged 23–51 years Nurses: aged 23–42 years All other groups, age not specified	No explicit measure provided	Self‐stigma associated with low education and reduced care‐seeking Reduced care‐seeking associated with lack of social support and cost of treatment/management Cultural influence on inaccurate knowledge (e.g., superstition, contagiousness) associated with more stigmatization Concealment positively associated with stigma and belief of heredity as cause Higher levels of stigma are positively associated with more physical experiences of disease Social and self‐exclusion Discrimination Reduced life opportunities	Social Self Associative Structural
Tiamkao et al. (2013), Thailand	Methodology: Quantitative Cross‐sectional survey Sample: 1,000 randomly selected adults (500 municipal [urban], 500 rural) Gender: 445 male (195 rural), 555 female (305 rural) Age: Mean age: 48.7 years (rural), 34.4 years (urban)	No direct measure of stigma but assesses related aspects of knowledge, attitudes, and practices (KAP)	Low levels of knowledge regarding epilepsy overall In general, nonmunicipal people (Rural) in this study were older, had more married persons, and lower levels of education and income, than municipal people (Urban) More rural cultural influence on inaccurate knowledge (e.g., “spiritual possession,” “hereditary”) associated with more stigmatization Concealment higher for rural than urban More social exclusion, reduced life opportunities and discrimination increased for rural vs. urban	Social Self Associative
San‐Juan et al. (2015), Mexico	Methodology: Quantitative Cross‐sectional survey Sample: 162 rural adults (epidemiology survey) 33 (of the 162 rural adults) with seizure history or epilepsy (subsequent survey) Gender: Not provided Age: 18 years and older	No direct measure of stigma but assesses related aspects of knowledge/beliefs, reactions, and discrimination	Most (76%) did not attempt to conceal epilepsy Most (96%) reported experiencing epilepsy stigma More than half did not seek physician care Most (70%) reported inaccurate knowledge which appeared to be influenced by culture (e.g., “divine punishment”), associated with reduced professional care‐seeking and increased traditional care‐seeking	Social Self Structural
Kartal et al*. *(2016), Turkey	Methodology: Quantitative Cross‐sectional survey Sample: 500 (351 urban, 149 rural) healthy hospital visitors Gender: 247 male, 253 female Age: 18–72 years (mean age 34.09 years)	No direct measure of stigma but assesses related aspects including knowledge/familiarity, and attitudes	Fewer rural than urban were familiar/knowledgeable about epilepsy Rural had more negative attitudes and reduced life opportunities versus urban Across Rural/Urban Predictors of negative attitudes were being female, lower education, and living rurally Negative attitudes mainly about objecting to marrying a person with epilepsy and perception of people with epilepsy being unable to live alone Main reasons for negative attitudes stemmed from misconception of epilepsy as “a dangerous and lifelong disease”	Social Self
von Gaudecker et al. (2017), India	Methodology: Qualitative Semi‐structured Interviews, observations, and field notes Sample: 16 rural adults (6 diagnosed with epilepsy, 8 family caregivers, 2 traditional healers) Gender: 6 female (diagnosed with epilepsy) Not provided for family caregivers and traditional healers Age: 20–63 years (for the 6 diagnosed with epilepsy) Not provided for family caregivers and traditional healers	No explicit measure of stigma provided.	Authors noted stigma during recruitment where women of marital age refused participation Epilepsy‐related stigma increased “physical psychological, and emotional struggles” Cultural influence on inaccurate knowledge and false beliefs (e.g., (“divine curse”) associated with more stigmatization and seeking alternative/traditional care (vs. medical) Social and self‐exclusion associated with physical manifestations of the disease Concealment Felt somewhat deserved of their “fate” and should “suffer in silence” due to burden on family Remained hopeful to be happy, independent, happily married, and have healthy children	Social Self Associative
Allard et al. (2017), United Kingdom	Methodology: Quantitative Cross‐sectional survey Sample: 46 rural adults with epilepsy Gender: 29 male, 17 female Age: Over 18 years (mean age 37.8 years)	Jacoby's 3‐item Stigma Scale	Overall, 65% of total Emergency Department (ED) use was for epilepsy‐related issues ED use positively associated with experience of more seizures and living in socially deprived area Stigma, depression, medication management, knowledge (“related to social and medical aspects of the condition”) were not significantly related with ED use Living in a more socially deprived area was associated with more frequent ED use for seizures	None
Dementia and Alzheimer's Disease (*n* = 7)				
Arai et al. (2000), Japan	Methodology: Mixed Methods Cross‐sectional ‐ Zarit Burden Interview (ZBI) (Japanese version) Semi‐structured interviews (SSI) Sample: ZBI: 70 rural family caregivers of “elderly in need of care” SSI: 7 (of the 70) rural family caregivers Gender: 25 male, 45 female Age: Mean age 59.5 years (*n* = 70)	No explicit measure provided.	Note: This study examines stigmatization of formal public service use, not necessarily specific to dementia 70% of the “elderly in need of care” were diagnosed with dementia Service nonusers were more likely to be concerned about the opinions of others Caregivers of more dependent elderly used more services Some caregivers reported negative judgment of others associated with service use, both upon themselves (as not capable) and upon the elderly being cared for (as severely impaired)	Social Self
Morgan et al. (2002), Canada	Methodology: Qualitative Focus groups (3) Sample: 22 rural formal (13) and family (9) caregivers Gender: 1 male, 8 female (family caregivers only) Formal caregiver gender not provided Age: Not provided	No explicit measure provided	Stigma associated with dementia had both direct and indirect impacts on reducing caregivers’ use of services Identified barriers to service use associated with stigma: rural lack of privacy, lack of awareness [knowledge], beliefs and negative attitudes Denial, shame, embarrassment, isolation/exclusion, concealment and “socially inappropriate behaviours common to dementia” were identified as stigmatic potential barriers to care‐seeking “[Dementia] is associated with mental illness, which has a long history of misunderstanding, fear, and stigma.” Service use identified as a form of “charity”, particularly among older females caring for their husbands, associated with guilt and further social isolation Generational increase in awareness [knowledge] identified as possibly having a positive effect	Social Self
Cahill et al. (2008), Ireland	Methodology: Mixed Methods Cross‐sectional survey Focus group Sample: Survey: 300 general practitioners (general practitioners) (237 urban, 63 rural) Focus group: 7 (of the 63) rural general practitioners Gender: Survey: Gender not provided Focus group: (5 male, 2 female) Age: Not provided	No explicit measure provided.	Both Rural & Urban General Practitioners (general practitioners) (survey): Stigma a key issue in delayed diagnoses; general practitioners reasons of therapeutic nihilism, lack of time/confidence/education and not looking enough for the disease; Patient reasons for patient/family denial or shame, dementia as normal ageing, and therapeutic nihilism) Rural general practitioners (Focus Group) Long‐term familiarity with patients as a potential barrier to noticing minor dementia‐related changes over time, until an obvious time of crisis occurs Stigma and scepticism about benefits to early diagnosis; general practitioners may not want to label a person until approached by family or a time of crisis General practitioners felt “geographically disadvantaged” regarding access to diagnostic services and perceived themselves as “more actively involved in dementia Screening than their urban counterparts”	Social Self Health Professional Associative Structural
Stansbury et al. (2010), United States	Methodology: Qualitative In‐depth semi‐structured interviews in response to an Alzheimer's Disease vignette Sample: 9 African American Baptist clergymen presiding over rural churches Gender: 9 male Age: 36 to 58 years (median age 41 years)	No direct measure of stigma provided but assesses related aspects including: Knowledge, beliefs, and attitudes	Most had vague but generally accurate knowledge about Alzheimer's Disease (Alzheimer's disease) and recognized importance of seeking professional medical advice Most admitted being uninformed about how people with Alzheimer's disease could be referred to various health care services Most acknowledged the importance for people with Alzheimer's disease to “retain some form of dignity” while adjusting to the disease (e.g., driving cessation) Most noted Alzheimer's disease as “fear‐provoking” due to progressive nature and incurability	Structural
Forbes et al. (2011), Canada	Methodology: Qualitative In‐depth interviews Sample: 17 rural caregivers (of 3 persons with dementia) (14 family, 3 personal support workers) Gender: Family caregivers: 5 male, 9 female Personal support workers: gender not provided Age: Not provided	No explicit measure provided	Rural living can offer community support that reduces stigma but lacks privacy which can increase stigma Social and self‐exclusion are often associated with dementia‐related behavioural or physical occurrences Dependency on others for care and lack of involvement in decision‐making results in disempowerment and exclusion Reduced life opportunities for people with dementia and their families who are often “treated differently”	Social Self Associative Structural
Burgener et al. (2015) (Parts 1 and 2), United States	Methodology: Mixed Methods Descriptive, longitudinal mixed methods prospective design (at 6‐month intervals over 18 months) Demographic data sheet Clinical Dementia Rating Scale (CDR) Mini‐Mental State Exam (MMSE) Stigma Impact Scale Several measures for quality of life outcomes (e.g., The Geriatric Depression Scale, Revised Memory and Behavior Problems Checklist, Rosenburg's Self Esteem Scale, Duke Social Support Index, 5‐item health subscale from Medical Outcomes Trust SF−36 health survey) Structured interviews Sample: 50 adults recently diagnosed with Alzheimer's disease or other dementia (26 urban, 24 rural) 47 corresponding family caregivers Gender: Adults with Alzheimer's disease or other dementia: 24 male, 26 female Family caregivers: 12 male, 34 female Age: Adults with Alzheimer's disease or other dementia: 62–104 years (mean age 78.3 years) Family caregivers: 26–82 years (mean age 64.4 years)	Stigma Impact Scale (SIS) (Fife and Wright, 2000), revised for people with dementia (previously tested by Burgener and Berger, 2008)	Across Rural/Urban Some aspect of stigma was associated with all quality of life outcomes (e.g., depression, behavioural symptoms, physical health) Social rejection and isolation reported more by those with higher cognitive function Dependency on spouse for social involvement Concealment associated with those less well‐known to the person with dementia [or family] and with previous negative responses of others upon disclosure Perceived stigma stable over 18 months then decreasing trend Stigma decreased as age increased Rural vs. Urban At baseline, rural people with dementia and Alzheimer's disease reported lower levels of internalized shame than urban	Social Self
Hsiao et al*. *(2015), China	Methodology: Mixed Methods Cross‐sectional socio‐demographic survey Focus Groups (2 urban, 2 rural) with researcher‐observer notes Sample: 40 mental health providers (20 rural, 20 urban) Gender: 20 male (10 urban, 10 rural), 20 female (10 urban, 10 rural) Age: Mean age 33.5 years	No direct measure of stigma provided but assesses related aspects including: Knowledge, attitudes, and practices	Rural/Urban Differences Rural less experienced with dementia in general and were more underdiagnosed Rural more likely to consider cognitive issues as lower priority than physical issues Rural was less empathic, knowledgeable, and confident in dementia‐related counselling Rural expressed more difficulty communicating with patients/families about diagnoses and related frustration and stress Rural offered more life/culture‐focused advice for dementia treatment/management (vs. urban, more about sociality, diet, stress) Across both Rural and Urban Lack of education, training, and specialist care Health professional therapeutic nihilism and ageism Low awareness [knowledge] among family caregivers that dementia is a disease and not a normal part of ageing Social and self‐exclusion Discrimination Structural stigma exists regarding lack of systems, specialized teams, policies, or plans regarding government and hospital management for dementia	Social Self Associative Health professional Structural
Parkinson's Disease (*n* = 4)				
Klepac et al. (2007), Croatia	Methodology: Quantitative Cross‐sectional survey Parkinson's Disease Quality of Life Questionnaire Unified Parkinson's Disease [severity] Rating Scale (UPRS) part III (“with motor evaluation done during ‘on’ period”) Sample: 111 adults with Parkinson's disease (65 urban, 46 rural) Gender: 53 male (27 urban, 26 rural), 58 female (38 urban, 20 rural) Age: Mean age 66 years	Parkinson's Disease Quality of Life Questionnaire, with 4‐item stigma subscale) Other stigma‐related Parkinson's disease questions	Being rural was associated with poorer quality of life overall and worse social support, stigma, emotional well‐being, and bodily discomfort Both rural and urban reported increased stigma for late‐onset patients Both rural and urban reported increased stigma and poor social support for unmarried or those with motor issues Both rural and urban low nonmotor symptoms scores strongly associated with emotional well‐being, social support, cognition, stigma, communication, bodily discomfort	Social Self
Mshana et al. (2011), Tanzania	Methodology: Qualitative Focus groups (6) Semistructured interviews Sample: *N* = 62:28 rural adults with Parkinson's Disease (Parkinson's disease), 28 caregivers (25 family, 3 paid), 4 health workers, 2 traditional healers Gender: 32 male, 30 female Age: Individuals with Parkinson's disease: 45 to 94 years (26/28 older than 64 years) No age provided for caregivers, health workers, or traditional healers	No explicit measure provided.	All groups reported that Parkinson's Disease (Parkinson's disease) is strongly associated with lower quality of life in terms of “dependency, stigma, and social isolation” Cultural influence on inaccurate knowledge/beliefs (“witchcraft”) Stigmatization for people with Parkinson's disease from family and community, related to belief that Parkinson's disease is part of normal ageing Travel and high cost of treatment result in lack of professional medical care‐seeking and increase in seeking alternative traditional healers for care Social and self‐exclusion Dependency on others/Disempowerment	Social Self Structural
Wu et al. (2014), China	Methodology: Quantitative Cross‐sectional survey Non‐Motor Symptoms Scale Parkinson's Disease Quality of Life Questionnaire (Parkinson's disease) (Chinese version) Unified Parkinson's Disease Rating Scale Part III Modified Hoehn and Yahr Staging Minimental State Examination Sample: 649 adults diagnosed with Parkinson's Disease (418 urban, 231 rural) Gender: 365 male, 284 female Age: 19–83 years (mean age 61.7 years)	Parkinson's Disease Quality of Life Questionnaire, with 4‐item stigma subscale) Other stigma‐related Parkinson's disease questions	Across both rural and urban Increased stigma for late‐onset Parkinson's disease Increased stigma and poorer social support for those single/divorced/widowed or having motor difficulties and nonmotor symptoms Poorer quality of life associated with being female, more advanced disease stage and duration Being female was associated with poor emotional well‐being and bodily discomfort Rural/Urban Differences Rural indicated a poorer overall self‐perceived quality of life and poorer scores of emotional well‐being and bodily discomfort, especially among females	Social Self
Kaddumukasa et al. (2015), Uganda	Methodology: Quantitative: Cross‐sectional survey Sample: 377 adults (177 urban, 200 rural) Gender: 123 male, 254 female Age: 18–85 years (median age 34 years)	No direct measure of stigma provided but assesses related aspects including knowledge and attitudes	Overall, over half reported accurate knowledge about causality however, rural more frequently did not identify the brain being associated with Parkinson's disease Overall, just over one‐third believed people with Parkinson's disease could still be employed Rural were less accurate in identifying tremors and body stiffness as main Parkinson's disease symptoms	Structural
Machado Joseph Disease (*n* = 1)				
Paúl et al. (1999), Portugal	Methodology: Qualitative In‐depth interviews and field notes (subsequent to Machado Joseph Disease (MJD) group information meeting, group interviews, psychosocial evaluations, and genetic testing) Sample: 25 rural adults identified as at risk for MJD (Group meeting *n* = 25; Psychosocial evaluations 16/25, 14 of which underwent genetic counseling, and 12 of whom were interviewed) Gender: Interviewees: 6 male, 6 female (asymptomatic at risk: 1 male, 5 female; MJD: 5 male, 1 female) Age: Interviewees: asymptomatic at risk, 30 to 66 years (mean age 43); MJD, 59 to 71 years (mean age 67 years)	No explicit measure provided.	Most reported having no knowledge about Machado Joseph Disease (MJD) prior to study Most reported spending excessive time and money care‐seeking for symptoms and lacking accurate diagnoses Many reported being labelled as “drunk” due to ataxia For those without MJD but at risk for developing, most reported expectations of family to care for them if/when incapacitated	Social Self Structural
Traumatic brain injury (*n* = 2)				
Farmer et al. (2003), United States	Methodology: Quantitative The Hesitation Scale “selected portions of the Living Life After traumatic brain injury (LLATBI) scale including demographics, injury variables, community integration indicators, and quality of life ratings” Mini‐Mental State Exam Substance abuse measures Quality of life scale Social Support Scale Sample: 56 adults with Traumatic Brain Injury (traumatic brain injury) (15 urban, 22 town/suburban, 19 rural) Gender: 29 male, 27 female Age: 18–74 years (mean age 38 years)	No direct measure of stigma but assesses related aspects including: Perceptions about support seeking, participation, satisfaction, quality of life	Rural/Urban Differences Urban reported more negative attitudes and beliefs compared to town or rural Living rurally was significantly associated to better quality of life and seeking social support (vs. urban or town dwellers) Across both Rural and Urban Hesitation to seek social supports associated with being divorced or separated Social and self‐exclusion Intolerance, disempowerment, and reduced life opportunities	Social Self
Alston et al. (2012), Australia	Methodology: Qualitative In‐depth interviews and interviewer notes Sample: 11 rural women with Traumatic Brain Injury (traumatic brain injury) Gender: 11 female Age: 24–66 years	No explicit measure provided.	Concealment Social and self‐exclusion Fear of negative reactions from others	Social Self
Neurological disease in general (*n* = 1)				
Birbeck et al. (2002), Republic of Zambia	Methodology: Quantitative Semistructured cross‐sectional survey Sample: 80 nonphysician primary healthcare workers (9 urban, 64 rural, 7 “neither urban nor rural”) Gender: 59 male, 21 female Age: Not provided	No explicit measure provided.	Regarding neurological diseases in general: Across rural and urban (data not parsed) Inadequate training/knowledge to care for people with neurological disorders in general (e.g., in spite of 66% reporting encounters with seizures, only 33% reported feeling adequately trained or experienced in this area) Perceived patient barriers to seeking, accessing, and continuing care: Social stigma Long waiting times Distant referral site Physician/specialist care, testing and drug costs (“discriminatory healthcare policies”) Fear of dying in tertiary care and associated costs Traditional beliefs and lack of community health‐related knowledge	Social Structural

Note

Verbatim information marked within quotations.

Neurological diseases of focus in these studies were: epilepsy (*n* = 21), Alzheimer's disease or other dementia and (*n* = 7), Parkinson's disease (*n* = 4), traumatic brain injury (*n* = 2), Machado‐Joseph disease (*n* = 1), and neurological disease in general (*n* = 1) (Figure 4). The majority of studies focused on stigma related to epilepsy (21/36), almost half of which were conducted within the continent of Africa (10/21).

Of the 36 studies, there were 20 quantitative, 10 qualitative, and six mixed methods. Most of the studies specified the involvement of both males and females in their sample (31/36) and of these, 19 considered sex in their analyses regarding stigma‐related outcomes. Ten of these found no significant differences in stigma‐related outcomes for males versus females; however, one of these (Rafael et al., 2010) reported that significantly more males never married compared to females. Of the nine studies that found significant differences, most (7) reported better stigma‐related outcomes for males compared to females.

The majority of included studies (24/36) provided no definition of rural; instead the authors simply stated or implied the area and/or sample under study was rural. Standardised definitions were used in 7/36 studies, such as census data or other government statistics and maps, or national agency resources. Four of the 36 studies stated that the location and/or sample was rural and provided population size, often with the relative distance from an urban centre.

Over half of the studies were rural‐focused (20/36); 16/36 included urban participants, 15 of which provided at least some rural‐urban comparisons and one in which rural and urban data were not reported separately. Most of these comparative studies reported rural‐urban differences in stigma‐related findings (13/15). The findings of the 15 studies that included rural‐urban comparisons are reported briefly within each section of the findings, and then summarised at the end of the Findings section.

All five types were identified within the 36 included studies: social (33), self (25), structural (14), associative (13), and health professional (3). While some studies measured stigma with scales (7), many involved quantitative assessment of stigma‐related concepts (such as knowledge, attitudes, practices, beliefs) (24), qualitative analysis (15), or combinations thereof. Although different methods to assess stigma made comparison difficult, they provided a rich data set that encompassed a broad scope of findings regarding stigma‐related concepts.

### 3.2 Factors influencing stigma

#### 3.2.1 Knowledge, familiarity, beliefs

Knowledge refers to an accurate understanding of the neurological disease, familiarity refers to knowing someone or having had experience with the neurological disease, and beliefs refers to perceptions or opinions about the neurological disease. In general, the influence of knowledge, familiarity and beliefs on stigma were often interrelated. Most of the studies (26/36) reported stigma‐related outcomes that were influenced by knowledge, familiarity, and/or beliefs about the neurological disease being studied. Within these studies, all five types of stigma were identified.

Overall, a lack of accurate knowledge about the neurological disease was associated with increased stigma (24/26). While the influence of familiarity on knowledge and stigma were mixed, several of these studies reported effects of familiarity and beliefs on knowledge about the disease that in turn, informed stigmatising attitudes and behaviours. Across 15 studies, beliefs (particularly cultural) were associated with less accurate knowledge and increased stigma. For example, Mshana, Dotchin, and Walker (2011) reported that high familiarity, a lack of knowledge, and inaccurate beliefs about the cause of Parkinson's disease (e.g., “witchcraft” or “normal ageing”) were related to increased stigmatisation.

Similar findings were reported by Bain, Awah, Takougang, Sigal, and Ajime (2013) regarding epilepsy among rural community members in the Republic of Cameroon with reports of high familiarity, a lack of knowledge and incorrect beliefs of cause (such as “demonic possession”) being associated with more stigmatisation, particularly among older, less educated females. Kim et al. (2003) reported similar findings of high familiarity, a lack of knowledge, and influence of cultural beliefs associated with more stigmatisation for older, less educated females in their epilepsy study of rural adults in Korea. However, Osungbade and Siyanbade (2011) focussed on rural adults in Nigeria regarding epilepsy and reported similar high familiarity, accurate knowledge, and beliefs associated with reduced stigma, and reported that females (particularly over age 30 years) were more knowledgeable than men.

In general, studies that involved urban‐rural comparisons reported less knowledge about the neurological disease among rural dwellers and/or healthcare providers; this in turn was associated with increased stigma in rural areas (Birbeck, Chomba, Atadzhanov, Mbewe, & Haworth, 2006; Deresse & Shaweno, 2016; Hsiao, Liu, Xu, Huang, & Chi, 2015; Kaddumukasa et al., 2015; Kartal & Akyildiz, 2016; Tuan, Coung, Allebeck, Chuc, & Tomson, 2007).

#### 3.2.2 Rurality

Rural was also identified as a factor influencing stigma. All five types of stigma were identified within the six studies that remarked on the interconnectedness among people living in rural areas, which reportedly had mixed effects on stigma. Morgan, Semchuk, Stewart, and D'Arcy (2002) and Forbes, Ward‐Griffin, and Kloseck (2011) explored the experiences of rural families caring for people with dementia. Forbes et al. (2011) found that rural living offered a close‐knit, supportive community that could reduce stigma. However, Forbes et al. (2011) and Morgan et al. (2002) reported that a lack of privacy experienced by rural dwellers sometimes led to increased stigma. Of the 15 studies that explored differences in stigma‐related outcomes between rural and urban participants, only three reported more positive findings for rural than urban (Burgener et al, 2015; Farmer, Clark, & Sherman, 2003; Youssef et al., 2009) and one that reported both better and worse stigma‐related findings among rural compared to urban (Yang et al., 2011).

### 3.3 Stigma‐related themes

#### 3.3.1 Concealment

Concealment refers to attempts to hide the neurological disease to avoid being stigmatised. With the 11 studies reporting concealment associated with stigma, all five types of stigma were identified: social (11), self (11), structural (4), associative (3), and health professional (1). Studies focused on epilepsy (7), Alzheimer's disease or other dementia (3), and traumatic brain injury (1).

Most of the 11 studies focussed solely on rural populations (8). In general, these studies reported similar findings of attempts to hide the neurological disease. Of these, two studies found an association between attempts to conceal and the more physical nature of the disease. Guo et al. (2012), who interviewed rural adults in China, found that concealment of epilepsy was associated with not only experiencing physical manifestations of the disease, but also with the belief that epilepsy is hereditary. This finding is similar to that of Morgan et al (2002) who conducted focus groups in their Canadian study with rural caregivers of people with dementia. They found stigmatic effects of shame and embarrassment associated with the disease, specifically in terms of social behavioural issues, that resulted in concealment and was identified as a potential barrier to care‐seeking. San‐Juan et al. (2015) however, found that even though almost all adults with epilepsy (94%) who participated in their study in rural Mexico reported having experienced epilepsy‐related discrimination, only 24% reported attempts to conceal the disease.

Three of the 11 studies reporting concealment associated with stigma provided urban‐rural comparisons, with mixed results (Burgener et al., 2015; Tiamkao, Sawanyawisuth, Singhpoo, Ariyanuchitkul, & Ngamroop, 2013; Yang et al., 2011). For example, Burgener et al. (2015) conducted a longitudinal study to assess stigma among people with Alzheimer's disease or other dementia and found lower levels of internalised shame at baseline among rural participants compared to urban, in addition to rural participants (men in particular) being more accepting of disease concealment than urban. Tiamkao et al. (2013) found more concealment of epilepsy among rural participants in Thailand (who were also less educated, earned a lower income, older, and more likely married) versus urban participants.

#### 3.3.2 Social exclusion

Twenty‐three studies reported social exclusion (either by others or self‐isolation) associated with stigma. Within these 23 studies, all five types of stigma were identified: social (23), self (19), structural (10), associative (9), and health professional (2). The studies concerned epilepsy (15), Alzheimer's disease or other dementia (4), Parkinson's disease (2), and traumatic brain injury (2).

Most (15/23) of the studies that reported findings of social exclusion focused solely on rural populations. Neni, Latif, Wong, and Lua (2010) found that most rural community members surveyed (77.9%) supported people with epilepsy being involved and socialising in the community. Other rural‐focused studies (10/15) reported associations between social exclusion and other variables. Five studies assessed stigma‐related aspects (e.g., knowledge, attitudes, beliefs, and practices) regarding epilepsy and reported a positive association between social exclusion and false beliefs about epilepsy causality (e.g., contagious or “madness”) (Bain et al., 2013; Mugumbate & Mushonga, 2013; Ojinnaka, 2002; Osungbade et al*.*, 2011; Tuan et al., 2007).

Three rural‐focused studies (two regarding Alzheimer's disease or other dementia and one epilepsy), reported a positive association between social exclusion and the physical and/or behavioural manifestations of the neurological disease (Forbes et al., 2011; von Gaudecker, Taylor, Keeling, Buelow, & Benjamin, 2017; Morgan et al., 2002). Klepac et al. (2007) reported similar findings among individuals with Parkinson's disease where higher levels of social support were strongly associated with having low nonmotor symptoms scores. Similarly, Guo et al. (2012) found that among people with epilepsy, the physical nature of the disease was associated with increased stigma in general and that self‐stigma (reportedly tied to self‐exclusionary practices) was associated with low education and reduced care‐seeking.

Of the eight urban‐rural studies that reported social exclusion, three reported urban‐rural differences (Burgener et al., 2015; Farmer et al., 2003; Tiamkao et al., 2013). For example, regarding epilepsy, Tiamkao et al (2013) found a positive association between social exclusion and being rural, whereas Farmer et al. (2003) found that experiences of social exclusion were similar across urban, subrural, and rural adults with traumatic brain injury.

#### 3.3.3 Disempowerment, discrimination, unequal life opportunities

Twenty‐one studies reported stigma‐related outcomes regarding disempowerment, discrimination, and/or unequal life opportunities. Within these 21 studies, all five types of stigma were identified, including social (20), self (14), associative (11), structural (9), and health professional (2). The studies were related to epilepsy (15), Alzheimer's disease or other dementia (2), Parkinson's disease (2), Machado‐Joseph disease (1), and traumatic brain injury (1).

Over half of the studies (11/21) that reported findings of disempowerment, discrimination, and/or unequal life chances focused on rural populations, most of which (8) were regarding epilepsy. For example, Guo et al. (2012) found most people with epilepsy had been discriminated against, even by distant family members. Employer discrimination was present, most of whom admitted that they would not hire a person with epilepsy Guo et al. (2012). McQueen and Swartz (1995) found similar outcomes among respondents who experienced dependency on others and had difficulty finding employment, other than jobs specifically designed for people with limited capabilities. Bain et al. (2013) reported that most community members surveyed admitted that they would not give equal employment opportunities to people with epilepsy, nor would they allow their children to associate with or marry a person with epilepsy. Bain et al (2013) also noted an association between discrimination and unequal life opportunities with inaccurate knowledge about the disease (Bain et al., 2013).

Neni et al. (2010) found that almost half of the participants surveyed reported that people with epilepsy should not participate in sports, driving, or marriage. Rafael et al. (2010) reported similar findings which included dependency on others and difficulty getting married. Difficulties getting married were experienced more among men than women, and more men than women with epilepsy were unmarried and more men than women expressed poorer chances of getting married (Rafael et al., 2010).

Findings of disempowerment, discrimination, and unequal life opportunities were also reported in studies focusing on neurological diseases other than epilepsy among in rural‐focused studies (3/11). For example, Forbes et al. (2011) found disempowerment and discrimination among people with dementia (and often their families) who were treated differently, lacked involvement in decision‐making about their care, and felt limited in terms of choices and control. Mshana et al. (2011) found similar results among adults with Parkinson's disease in rural Tanzania, as did Paúl et al. (1999) in rural Portugal among people at risk for Machado‐Joseph disease. Paúl et al found that those at risk of developing the disease had expectations of becoming dependent on their family members if and when they became incapacitated.

In general, rural–urban comparison studies across several countries reported intolerance, discrimination, and unequal life opportunities more often for rural populations versus urban (Attadzhano, Chomba, Haworth, Mwewe, & Birbeck, 2006; Birbeck et al., 2006; Deresse & Shaweno, 2016; Kartal & Akyildiz, 2016; Tiamkao et al., 2013; Yang et al., 2011). For example, Attadzhano et al., 2006 and Birbeck et al. (2006) found that rural participants were less tolerant than urban participants, and intolerance across urban–rural was associated with being younger and less educated. Other studies involving urban–rural comparisons among people with traumatic brain injury, dementia, or Parkinson's disease (3/7) reported intolerance, discrimination and unequal life opportunities. In addition to living rurally, Kartal and Akyildiz (2016) reported that across urban and rural participants, predictors of negative attitudes included being female and less educated.

#### 3.3.4 Healthcare systems, services, and providers

Nine studies reported stigma‐related outcomes regarding healthcare systems, services, and/or providers. Two subthemes (availability and accessibility, and healthcare provider competencies and values) were identified. Within these nine studies, all five types of stigma were present: social (8), self (7), structural (7), associative (4), and health professional (3). Within these studies, the neurological diseases of focus included epilepsy (3), Alzheimer's disease or other dementia (3), Machado‐Joseph disease (1), Parkinson's disease (1), and neurological disease in general (1).

##### Availability and accessibility

Eight studies reported stigma‐related outcomes regarding access and availability (rural: *n* = 4; rural‐urban: *n* = 4). Hsiao et al (2015) noted existing structural stigma in the lack of systems, specialised teams, policies, or plans regarding government, and hospital management for dementia. Cahill et al. (2008) conducted a cross‐sectional study of urban and rural general practitioners in Ireland, followed by focus groups with the rural practitioners. Stigma was identified as a key factor in delayed diagnoses, where rural general practitioners felt disadvantaged in terms accessing diagnostic services due to geographic location.

Several studies reported reduced care‐seeking and/or service use associated with a lack of available, timely access to affordable healthcare, services, and treatment (Birbeck & Munsat, 2002; Guo et al., 2012; McQueen & Swartz, 1995; Mshana et al., 2011; Paúl et al., 1999). Mshana et al. (2011) reported an additional association between delayed diagnoses and lack of medicalised treatment with increased use of traditional healers due to the belief their illness was due to a curse or witchcraft. The use of traditional healers was also increased due to a lack of locally available Parkinson's disease drugs and the associated costs, even if an individual did obtain a medical diagnosis. Only 2/28 individuals with Parkinson's disease were receiving westernised treatment, both of whom reported the drugs gave them enough relief to lead normal lives, when they could afford to buy them (Mshana et al., 2011).

Stansbury, Marshall, Harley, and Nelson (2010) explored the attitudes towards and knowledge about Alzheimer's disease among rural clergymen in the United States, whose congregation often opted to seek their advice for health‐related issues. Clergymen acknowledged that in spite of being able to offer advice on other health issues, they were largely uninformed about Alzheimer's disease‐related healthcare services and referral processes and yet were looked to be responsible for advising their members with the disease.

##### Healthcare provider competencies and values

Three studies reported findings regarding the capabilities and willingness of healthcare providers to provide adequate care, one regarding neurological diseases in general where urban–rural data were not reported separately (Birbeck et al., 2002) and two regarding dementia (Cahill et al., 2008; Hsiao et al., 2015). All three studies were conducted among urban and rural populations. In general, a lack of sufficient time, experience, training, education, and confidence among primary healthcare professionals to diagnose and treat neurological diseases were reported (Birbeck et al*.*, 2002; Cahill et al, 2008; Hsiao et al., 2015). For both urban and rural populations, this often resulted in delayed diagnoses or under‐diagnosis, where rural residents were underdiagnosed more frequently than urban (Cahill et al., 2008; Hsiao et al., 2015).

Other underlying reasons for delayed diagnosis or underdiagnoses attributed to healthcare professions in rural areas included: long‐term familiarity with patients as a potential barrier to noticing minor dementia‐related changes over time until more obvious changes occur, scepticism of benefits to early diagnosis, and avoidance of labelling a person with dementia until a time of crisis or approached by the family (Cahill et al., 2008). Hsiao et al. (2015 **)** also reported that relative to urban, rural health providers often perceived dementia‐related cognitive issues as lower priority than physical issues of other diseases. Moreover, rural providers were less empathetic, expressed greater difficulty communicating with patients and families about diagnosis, and offered more life and culture‐focused advice for dementia treatment and management.

### 3.4 Summary of rural–urban differences in stigma‐related outcomes.

Sixteen studies examined both rural and urban individuals including one in which rural/urban data were not reported separately (Birbeck et al., 2002), one with no findings of stigma‐related differences between rural and urban participants (Cahill et al., 2008), and 14 that reported rural–urban differences in stigma‐related outcomes. Of those 14, one study reported both a worse and a better stigma‐related outcome for rural compared to urban participants (Yang et al., 2011), and only three studies reported some better stigma‐related outcomes for rural versus urban participants (Burgener et al., 2015; Farmer et al., 2003; Youssef et al., 2009). Overall, 12/14 reported not only worse, but also more stigma‐related outcomes for rural versus urban.

Seven (7/12) of the studies that reported poorer stigma‐related findings for rural participants versus urban focussed on epilepsy. Compared to urban, rural was generally: less tolerant (Atadzhano et al., 2006; Birbeck et al., 2006); less familiar and less knowledgeable (Birbeck et al., 2006; Kartal & Akyildiz, 2016); more concerned with marriage prospects (especially younger adults), feasibility of treatment, and psychosocial issues (Yang et al., 2011); more negative in terms of attitudes (Kartal & Akyildiz, 2016; Yang et al, 2011); more likely to be influenced by cultural beliefs, more likely to conceal, be socially excluded, and discriminated against (Tiamkao et al., 2013); and more likely to experience a reduction in life opportunities (Deresse et al., 2016; Kartal & Akyildiz, 2016; Tiamkao et al., 2013). Tegegne and Awoke (2016) found that rural people with epilepsy were twice as likely to experience stigma in general compared to urban.

Only 2/8 epilepsy studies that reported urban‐rural differences found stigma‐related outcomes that were better for rural participants relative to urban. Youssef et al (2009) found that rural people experienced less stigmatisation in general than their subrural and urban counterparts. In addition to the poorer stigma‐related outcomes for rural versus urban participants noted above, Yang et al. (2011) also found that rural participants (men in particular) were more accepting of disease concealment versus their urban counterparts.

Of the studies that involved rural and urban participants focusing on other neurological diseases (*n* = 8), one did not report rural–urban data separately (Birbeck et al., 2002), and one found no differences (Cahill et al., 2008). Four (*n* = 3 Parkinson's disease; *n* = 1 dementia) found worse stigma‐related outcomes for rural participants versus urban. Rural individuals, especially females, with Parkinson's disease reported poorer quality of life, poorer emotional well‐being, and greater bodily discomfort (Klepac et al., 2007; Wu et al., 2014) and rural adults in general were less knowledgeable about the disease (Kaddumukasa, 2015). Regarding dementia, Hsiao et al. (2015) found a range of stigma‐related issues among rural versus urban general practitioners in China. Specifically, rural practitioners were less experienced, knowledgeable, and empathic, more concerned with physical than cognitive issues, and reported more difficulty and frustration communicating with patients and families.

Two (2/8) nonepilepsy studies reported better stigma‐related outcomes among rural than urban participants. Farmer et al. (2003) found that urban participants had more negative attitudes and beliefs towards adults living with traumatic brain injury and living rurally was significantly associated with a better quality of life and seeking social support. Burgener et al. (2015) found that rural participants with Alzheimer's disease or other dementia, and their informal caregivers, had less “internalised shame” at baseline. Across both rural and urban, the authors reported a decline in overall stigma after 18 months and an inverse association between overall stigma and age.

## 4 DISCUSSION

This scoping review identified 36 peer‐reviewed studies that reported on the stigma associated with neurological disease in rural adult populations. Literature on this topic has significantly increased since 1992. These studies include people with neurological disease, their families and caregivers, community members, employers, people working in public positions and institutional spheres, and healthcare professionals. It should be noted that many studies referred less to “stigma” per se and more often explored stigma‐related factors and concepts in relation to neurological disease. For example, studies considered factors that influence stigma (e.g., knowledge, attitudes, practices, and beliefs) in terms of stigmatising behaviours such as the perpetuation of misinformation, prejudice, discrimination, exclusion, and false beliefs.

This scoping review has identified three key gaps in the existing literature. First, over half of the studies in this review focused on epilepsy and of these, over half were conducted within Africa. Second, many studies did not include gender as a variable in the analysis of stigma‐related outcomes. Third, further research is needed on stigma related to neurological disease from a cross‐cultural comparative research perspective (including how different cultural forces interact with rurality). Thus, to provide a more comprehensive understanding of the stigma‐related impacts of neurological disease in rural populations, it is recommended that future research be expanded to further investigate this social phenomenon as it relates to other neurological disorders beyond epilepsy and explore potential gender or cultural differences in stigma related to neurological disease, to inform, develop, and implement the most effective stigma‐reducing strategies.

Although the majority of studies provided no definition of rural and measures of stigma varied considerably, each of the five types of stigma (social, self, structural, associative, and health professional) were identified, and several factors that influenced stigma and stigma‐related themes emerged. Common factors that influence stigma included knowledge about the neurological disease, familiarity and beliefs about the disease, and rurality. In general, inaccurate knowledge and beliefs (of the participants, or reported by the participants in reference to others) and living rurally were associated with increased stigma, often related to lower levels of education, and highly influenced by cultural norms.

Of the 16 studies included in this scoping review that included both rural and urban populations in their studies, one study did not explore urban–rural differences in stigma‐related outcomes and one study found no differences. Of the other 14 urban–rural studies, three reported better stigma‐related outcomes for rural participants compared to urban and 11 reported worse stigma‐related outcomes for rural participants versus urban. However, although one of these studies (1/11) reported primarily worse stigma‐related outcomes for rural versus urban participants, the authors also reported that rural participants were more accepting of concealment compared to urban participants. While this finding could be interpreted as a positive outcome for rural versus urban, it is possible that it could indicate increased stigma for this population where concealment reflects a need or expectation to do so. Overall, inaccurate knowledge was highly influenced by cultural beliefs and rural populations were, in general, less familiar with and less knowledgeable about the neurological disease and individuals with neurological conditions experienced more stigmatisation than their urban counterparts.

While an influence of culture per se was not mentioned, the rural–urban study of Farmer et al. (2003) and two rural‐focused studies of Morgan et al. (2002) and Forbes et al. (2011) reported that the supportive social networks of rural societies could reduce stigma, while the latter two authors also referred to a lack of privacy in rural settings that could increase stigma. Morgan et al. (2002) also found that service use by family caregivers of people with dementia was at times perceived to be a form of charity, particularly among older females caring for their husbands, which increased their guilt and social isolation. Three urban–rural comparison studies found no differences in stigma for rural individuals compared to urban individuals although two of these studies reported other relevant findings. Klepac et al. (2007) found that living rurally was associated with poorer quality of life and emotional well‐being for people with Parkinson's disease and Yang et al. (2011) reported more negative attitudes in general towards epilepsy among rural participants compared to urban.

Although rural and urban environments vary around the world, there are some features common to each regardless of geographical location. For example, Adams (2008) suggests that relative to urban, rural residents are often more associated with an agrarian history, less heterogeneous, more traditional, and less progressive on average, and less touched by modernity in general (as cited in Segaert, 2008, p.15). The studies included in this review were conducted in rural areas from around the globe (Figure 3) across different locales and cultures. Several of these studies involved populations that clearly remained more rooted in and attached to their local cultures and traditions, where beliefs about illness were often reported as “hexes” or “divine retribution” which increased stigma.

Health‐related structural stigma existed across the studies included in this review. Corrigan (2004) defines structural stigma as policies that limit the opportunities of, or bring about consequences for, stigmatised people that further perpetuate their stigmatisation. The limited access to available, affordable western‐medicine based healthcare services and trained, educated, and experienced healthcare professionals for rural adults with neurological disease appeared to be an inherent fact of life for most of the rural participants in this review. In addition, western medicine was often referred to by rural study participants as out of reach compared to non‐western traditional medicine where Indigenous healers were more readily available, familiar, accessible, and affordable. This is an important finding that warrants further exploration, as it may point towards a need for policy and interventions at the local community level to expand the range of accessible and affordable services. Furthermore, if people with neurological diseases are seeking and are able to access more care from traditional healers, there may be an opportunity for collaboration with traditional healers to identify effective stigma‐reduction strategies.

While findings of healthcare provider stigma were present within only three studies, the issue plays a key role in disease‐related stigma. The subjective opinions and beliefs of healthcare providers reportedly influenced their willingness to diagnose, resulting in delayed and under‐diagnoses; where urban–rural comparisons were made, this occurred more often among rural populations. For example, some practitioners were admittedly sceptical about the benefits of diagnosing an individual with dementia until a time of crisis and some reportedly placed a lower priority on cognitive versus physical issues.

Rommelfanger et al. (2017) speaks to the concept of healthcare professional stigma in practice in terms of functional neurological disorders (i.e., neurological symptoms with a psychiatric cause). The authors describe these patients as representing a “risk” for some healthcare providers due to extra time required for routine checks, and at times a lack of confidence in adequate disease expertise and resources for care. Such issues are relevant to neurological disease in general, particularly within rural populations with limited access to specialists. Sometimes health practitioners are resistant to diagnosing and labelling a person with dementia due to the stigma related to the label. Cahill et al. (2008) found that among general practitioners included in their study, stigma played a key role in delayed diagnosis of dementia. A dementia diagnosis could be termed a “double‐edged sword,” with both benefits and detriments. For example, while most general practitioners understand the importance of timely diagnoses, they are often aware of the stigmatising effects of diagnosing and labelling a person with dementia. Previous research has identified stigma as one of several factors that influence a practitioner's decision to diagnose dementia including disease severity, existing supports and services, confidence in their ability to adequately diagnose, personal beliefs, and existing societal and cultural norms (Low, McGrath, Swaffer, & Brodaty, 2018).

Some studies in our review found a positive association between the visible, physical nature of the neurological disease and the degree of stigmatisation. Scambler (1998) refers to illnesses with highly visible, obtrusive symptoms as being highly stigmatised. This is well‐supported within the literature across various types of diseases, along with the implications of forced or unintentional disease disclosure including psychological distress and reduced care‐seeking, for both the person with the disease and their families (Brener, Callander, Slavin, & de Wit, 2013; Maffoni, Giardini, Pierobon, Ferrazzoli, & Frazzitta, 2017; Phelan, Bromet, & Link, 1998; Scholl & Sabat, 2008; Stutterheim et al., 2012).

In addition to the association between concealment and the visibility of the neurological disease symptoms, concealment was associated with the stigma attached to what people believed to be the etiology of the disease. For example, neurological diseases were more stigmatised (resulting in more attempts to conceal) if they were thought to be hereditary, contagious, evidence of madness, or divine punishment. Attempts to conceal were often related to exclusionary practices, which were also often associated with misbeliefs about causality and the visibility of disease symptoms. Furthermore, concealment was associated with a reduction in care‐seeking and inadequate treatment and disease management. The effects of health‐related stigma on concealment has been supported in the literature, where even anticipated stigma increases attempts to conceal the disease. For example, Cook, Germano, and Stadler (2016) explored the association between stigma and concealment among a group of American adults with multiple sclerosis and found that anticipatory stigma was a strong predictor of concealment and that data trends indicated concealment was associated with delays in doctor visits and treatment therapy (Cook et al., 2016).

Findings of concealment and exclusionary practices in our review were also related to findings of disempowerment, discrimination, and unequal life opportunities. In general, findings included dependency on others for care and financial management, a lack of being involved in decision‐making about care, discrimination in terms of employability, marriageability, capacity to drive, and participate in sports. Marriage discrimination was reported in one epilepsy study as worse for males than females, however, no conclusions were drawn as to why (Rafael et al., 2010). Previous research supports these stigmatic effects of epilepsy in terms marriageability; however, the differential impacts for males versus females in general were mixed. Mixed findings could be influenced by culture, which appeared to play a role, especially in contexts of arranged marriages. A review of the literature on arranged marriage prospects among people with epilepsy in India found that females were impacted more negatively than males (Singh et al., 2016). According to the World Health Organization (2018), both China and India currently permit epilepsy as a valid reason to deny marriage. Perhaps people with epilepsy or other neurological disease are perceived as less employable and more likely to produce children with similar diseases.

In rural–urban comparison studies included in this review, rural populations in general reported either similar or more intolerance, discrimination, and unequal life chances relative to their urban counterparts. Yang et al. (2011) found that women more frequently than men reported that stigma of epilepsy was worse than stigma of other chronic diseases. However, compared to urban participants, rural residents were more negative towards epilepsy in general, more concerned about marriage prospects (especially younger participants), and more accepting of concealment (especially males) (Yang et al., 2011).

Differences in measures used to assess stigma existed across the studies included in our review. Future quantitative research to examine stigma associated with neurological disease might utilise a standardised measure such as the Stigma Scale for Chronic Illness (SSCI) developed by Rao et al. (2009). The SSCI is a nonillness‐specific stigma measure developed as part of a study regarding health‐related quality of life for people with neurological disorders. Consistent use of a validated measure in future research of health‐related stigma of neurological disease in rural (and urban) populations would allow for more accurate comparisons of differences on various levels. However, it is noteworthy that such a scale may not account for differences in particular contexts and across cultures. Cultural context clearly plays a role in stigma and most likely “methods of cultural epidemiology” (Weiss, Jadhav, Raguram, Vounatsou, & Littlewood, 2001, p.72) are required, with locale‐specific approaches that may be effective.

In addition, although most of the studies included in this review explicitly stated the number of males and females included in samples, gender was often not included as a variable in analysis of stigma‐related outcomes. Approximately half of the studies that considered sex in the analysis of stigma‐related outcomes found no differences; however, most of the studies that found differences were regarding epilepsy and were generally less favourable for females compared to males. Including gender‐based analysis in future research is important to explore potential differences in the ways in which stigma is experienced, and in the ways in which stigmatisation occurs, to develop and implement the most effective stigma‐reducing strategies.

In general, across all neurological diseases, stigmatising processes were involved in outcomes of discrimination, isolation, disempowerment, reduced care‐seeking, late diagnosis, and poor disease management. There is a paucity of research regarding stigma of certain neurological diseases among rural populations across the globe, with the exception of epilepsy studies conducted in rural areas in Africa. The important role of health‐related stigma in increasing the burden of illness for adults with neurological disease and their families is reflected in the recent study findings of the Canadian Dementia Priority Setting Partnership (Canadian Dementia Research Priorities, 2017). This study was designed to include the insight and experience of Canadians living with dementia and their families, friends, caregivers, healthcare providers, and others to identify the 10 most important dementia‐related areas on which research should focus moving forward (Canadian Dementia Research Priorities, 2017). Stigma associated with dementia and mental health issues was identified as the number one priority.

The following limitations of this scoping review warrant comment. The search strategy was limited to studies published in English language only, from 1992 forward, which could limit the generalisability of the findings. Also, grey literature (i.e., unpublished or nonpeer reviewed; see itemised list within Table 1) was not included, which could introduce potential for publication bias. Furthermore, as this is a scoping review and not a systematic review, the authors opted not to conduct a critical appraisal of study quality. Lastly, the authors did not attempt to address cross‐cultural comparative differences in the literature, which is a limitation of this review.

## 5 CONCLUSION

This scoping review highlights both important gaps in the existing literature, and recommendations for future research regarding stigma related to noncommunicable neurological diseases in rural areas. Three main gaps identified were limited attention to stigma related to neurological diseases other than epilepsy, few cross‐cultural comparisons of stigma related to neurological disease, and the inclusion of gender as a variable in the analysis of stigma‐related outcomes in only half of the reviewed studies. In addition, several key themes associated with stigma and stigma‐related concepts were identified.

The body of evidence synthesised in this review contributes to the knowledge available to policy makers, program planners, healthcare providers, and researchers. This knowledge can be used to inform future research and for the development of relevant, effective policy and intervention strategies designed specifically for rural areas, such as public awareness campaigns, to address the key issues and contributing factors that exist for this population. The overall goal of developing such strategies is to improve the lives of rural adults living with neurological disease and their families by reducing health‐related stigma and its negative consequences including exclusionary practices, reduced care‐seeking and service use, late diagnoses, and nonadherence to disease management and treatment strategies.

## CONFLICT OF INTEREST

No competing financial interests exist.
